# Synthesis of Amorphous InSb Nanowires and a Study of the Effects of Laser Radiation and Thermal Annealing on Nanowire Crystallinity

**DOI:** 10.3390/nano8080607

**Published:** 2018-08-09

**Authors:** Zaina Algarni, Abhay Singh, Usha Philipose

**Affiliations:** Department of Physics, University of North Texas, Denton, TX 76203, USA; zainaalgarni@my.unt.edu (Z.A.); abhaysingh@my.unt.edu (A.S.)

**Keywords:** amorphous *InSb* nanowire, space-charge-limited current (SCLC), Raman spectroscopy

## Abstract

Although various synthesis and characterization strategies have been employed for the synthesis of crystalline nanowires, there is very little work done on development of low-dimensional amorphous semiconductors. This paper presents a simple strategy to grow amorphous InSb (*a*-InSb) nanowires (NWs) in a chemical vapor deposition (CVD) system. The NWs were grown on Si substrate coated with indium film and the lack of crystallinity in the as-grown stoichiometric NWs was ascertained by Raman spectroscopy and electron transport measurements. A model proposed to explain the amorphous NW growth mechanism takes into account the fact that NW growth was carried out at the high temperature ramp-up rate of 75 ${}^{\circ }$C/min. This high rate is believed to affect the growth kinematics and determine the arrangement of atoms in the growing NW. Raman spectrum of the as-grown sample shows a broad peak around 155 cm${}^{-1}$, indicative of the presence of high density of homopolar Sb-Sb bonds in the amorphous matrix. It was also found that high intensity laser light induces localized crystallization of the NW, most likely due to radiation-stimulated diffusion of defects in *a*-InSb. The nonlinear trend of the current-voltage characteristics for individually contacted *a*-InSb NWs was analyzed to prove that the non-linearity is not induced by Schottky contacts. At high bias fields, space charge limited conduction was the proposed electron transport mechanism. Post-growth annealing of the as-grown *a*-InSb NWs was found to be very effective in causing the NWs to undergo a phase transition from amorphous to crystalline.

## 1. Introduction

In the last decade or so, there have been several works on synthesis strategies and determination of novel properties of crystalline semiconductor nanowires (NWs) [1]. This intense research effort has been motivated by the fact that crystalline NWs have promising electronic, photonic and optoelectronic properties, making them ideal for a large number of applications. Their unique properties, as determined by their one-dimensional structure, make them attractive as sensors and in photovoltaic, and thermoelectric [1] applications. Of the semiconducting NWs, the III-V group of semiconductors are promising and of these indium antimonide (InSb) is particularly interesting because it has the smallest direct energy band gap (0.17 eV at room temperature) [2], and very high electron mobility (77,000 cm${}^{2}$V${}^{-1}$s${}^{-1}$) [3]; making it an ideal material for use as field effective transistors and long-wavelength detectors [4,5]. Crystalline InSb (*c*-InSb) NWs have been synthesized by various techniques such as pulsed electrodeposition technique [2], pulsed-laser chemical vapor deposition [6], molecular beam epitaxy [7,8], metal organic vapor phase epitaxy [9], and chemical vapor deposition [10,11]. In most cases, growth of *c*-InSb NWs follows the metal-catalyst assisted vapor-liquid-solid (VLS) growth mechanism [1], and NW growth continues as long as In and Sb vapors are supplied to the molten alloy droplet.

Compared to crystalline semiconductor NWs, there have been limited investigations into amorphous NWs. This is most likely due to challenges in the synthesis of nanoscale amorphous materials [12]. The two main categories of amorphous semiconductors are: chalcogenide glasses which include elements of VI and their compounds, and the tetrahedrally bonded semiconductors which include Si, Ge, and amorphous III-V compounds [13]. Though several techniques are available for the characterization of *c*-InSb, experimental techniques that allow investigation of disordered amorphous InSb (*a*-InSb) are very limited. It is, therefore, important to develop an effective strategy for the synthesis of *a*-InSb NWs, where the amorphous phase and band structure can be tailored to enable the use of these NWs for energy storage and conversion applications [12].

Amorphous semiconductors are in general characterized by the absence of long-range order, but they preserve the short-range order that is characteristic of the crystal [14]. In the amorphous phase, the In-Sb bonds are longer (2.832 $\overset{˚}{A}$) and a large number of homopolar In-In and Sb-Sb bonds exist [14]. This lack of long-range order in amorphous materials has led to limitations in understanding their intrinsic electronic properties. There are reports on instabilities in measured current in this class of material, caused by defect distribution on surface as well as surface morphology and charge trapping [15,16]. The electrical properties depend not just on device geometry and nature of barrier between material and electrodes, but also on the level of disorder in the system. This causes amorphous materials of the same type to have very different properties. The other notable difference is in the electron transport mechanism in crystalline and amorphous materials. In crystalline materials, electrons are driven across a potential energy barrier that exists between the two metal contacts and the semiconducor as their Fermi levels align. However, in many amorphous materials, the current has a power law dependence on bias (electric fields) [17] and its magnitude is dependent on the defect type (Frenkel or Poole, charged or uncharged) and concentration.

Most III-V semiconductors like InSb have a tetrahedral bonding geometry involving In and Sb atoms, with a coordination number of four. This bonding geometry is maintained in *a*-InSb, but with slight elongation of the interatomic distances [14]. Because of the disorder in *a*-InSb, the band edges lose their sharpness and there exists a large density of localized states that tails into the energy band gap [18,19]. The localized electronic states causes carrier scattering, resulting in low carrier mobility, and also induces charge trapping [19,20]. Despite the fact that amorphous semiconductors have a high concentration of defects, the limited number of electrically active charge carriers causes them to be poor electrical conductors [13], with electrical resistivity values that are several orders of magnitude higher than the corresponding values in crystalline state. A similar difference is observed in the optical spectrum of amorphous materials, where the atomic disorder in the material causes the spectrum to be broadened with no sharp peaks. The X-ray diffraction pattern of amorphous semiconductors reveal no sharp spots, but shows intense diffusion rings or halos [13]. It has been reported [21] that the energy band gap of *a*-InSb ranges from about 0.4 to 0.65 eV, a four times increase compared to the energy band gap of *c*-InSb.

Synthesis of amorphous materials is generally low-cost and is carried out by relatively simple techniques. Due to absence of crystal quality constrains, such materials have structural flexibility and it becomes possible to construct materials with properties that cannot exist in the corresponding crystalline state [19]. It is also possible to heat-treat amorphous materials and convert them into a crystalline state, thus providing an economically viable route for the synthesis of crystalline semiconductors [12]. Since amorphous semiconductors are known to have high light absorption capability and high temperature coefficient of resistance, they are attractive in photoelectric and clean energy device applications [12]. Amorphous bulk materials or films are typically grown by two mechanisms: (i) lower the temperature abruptly so that the source melt is frozen in what is called the ‘melt-quenched amorphous phase’; (ii) growth from a vapor phase using techniques like thermal evaporation or a chemical vapor deposition (CVD) [13,14]. Previous reports include deposition of *a*-InSb film on various substrates by techniques such as flash evaporation on a cold substrate [21] and by sputtering at low temperature [22]. The studies validate the increases in energy band gap of *a*-InSb film from 0.17 eV up to 0.65 eV, and a significantly higher electrical resistivity. In terms of low-dimensional *a*-InSb structures, there are reports on the fabrication of high density near amorphous InSb NW arrays by electrodeposition method [23], where the amorphous nature of the NWs is attributed to three factors: (i) high concentration of the deposition solution, (ii) high concentration of the complexing agents, and (iii) high deposition voltage. Under these circumstances, the high concentration ions have no choice but to follow the lower energy site to deposit and grow. In another work, an array of polycrystalline InSb NWs was fabricated by electrodeposition [24], followed by two annealing steps: at 150 ${}^{\circ }$C and 450 ${}^{\circ }$C. The low temperature anneal enabled diffusion of In and Sb atoms in the InSb crystal and the high temperature anneal improved the crystalline quality of the NWs.

The process for synthesizing high quality *c*-InSb NWs in a CVD system has been well documented [10,11,25]. However, there has been no report on the growth of *a*-InSb NWs and hence no appropriate growth mechanism has been established. In this paper, we report on a strategy to grow *a*-InSb NWs in the CVD system. The lack of crystallinity in the as-grown NWs is attributed to the effect of a high temperature ramp-up rate. This work also highlights the effect of post-growth annealing treatment on the electronic and optical quality of the *a*-InSb NWs.

## 2. Materials and Methods

*a*-InSb NWs were synthesized in a CVD system, following the Vapor-Liquid-Solid (VLS) growth mechanism. A 200 nm thick In film was used as the seed layer to accomplish growth by a self-catalyzed process. Solid InSb crystals (Alpha Aesar, 99.99%) was the source material and the In coated Si substrates were placed face-down directly above the source. To ensure stoichiometry of the growing NWs, an extra source of Sb powder was placed near the InSb source. About 0.1 mg of sulfur powder was also placed at the low temperature end of the growth chamber. The introduction of sulfur served a two-fold purpose: (i) to ensure that any trace of oxygen in the growth chamber would be removed, thus inhibiting the growth of In and Sb oxides, (ii) sulfur is known to passivate III-V semiconductor surface. After the sample and source materials were loaded, the furnace was flushed with a mixture of H${}_{2}$ and Ar gas at (50 sccm) for 15 min, at room temperature. Earlier, the tube was annealed at 800 ${}^{\circ }$C under constant flow of (50 sccm) H${}_{2}$ gas for 1 h to prevent oxygen contamination. The source temperature was ramped up rapidly to 560 ${}^{\circ }$C at the fast rate of 75 ${}^{\circ }$C/min. *InSb* NW growth lasted for 90 mins at close to atmospheric pressure in a reducing environment of Ar: H${}_{2}$ gas at 50:50 sccm.

To study morphology and composition, the as-synthesized InSb NWs were characterized by a scanning electron microscope (SEM, Hitachi SU 1510, Dallas, TX, USA) equipped with energ- dispersive X-ray spectroscopy (EDX) and Raman Spectroscopy. Raman measurements were made on dispersed NWs at ambient temperature using a Nicolet Almega XR equipped with a 532-nm green laser. The size of the laser spot was ≈1 μm with a maximum power of 150 mW at 100× magnification. To study the electrical properties of the NWs, they were removed from the growth substrate and placed on a $p^{+}$Si substrate covered with a 200 nm thick SiO${}_{2}$ layer. The location of a single NW was recorded and two metal contacts (In/Au electrodes) were established at the two ends of the NW. The electrodes were patterned by standard photolithography process, followed by metal evaporation and lift-off. Three devices were fabricated, and each of them contained a single InSb NW (diameter about 200 nm and length about 15 μm) contacted by In/Au electrodes. A semiconductor parametric analyzer (Agilent Technologies B1500 A) was used to measure the electrical resistivity of the NW at room temperature.

## 3. Result and Discussion

The experimental design and growth parameters were optimized to ensure large aerial coverage of the Si substrate with InSb NWs. For a given thickness of the In film on the Si substrate, our experiments show that NW growth occurs in a very narrow temperature range. For an In film of thickness in the range of 200–300 nm, the growth temperature for stoichiometric InSb NWs was determined to be in the range of about 550–570 ${}^{\circ }$C, and the as-grown NWs had diameters in the range of 200–350 nm. For an ∼100 nm thick In film, a growth temperature of 550–570 ${}^{\circ }$C resulted in non-stoichiometric InSb NWs with very high In content and relatively larger diameter (∼150 nm). InSb NWs grown by the technique described in this paper had diameters larger than the thickness of the seed film (In) . One possible reason for this could be the fact that III–Sb NWs are reported to generally show large diameters [26] compared to other semiconductors. Moreover, the high temperature ramp-up rate, resulting in formation of large In islands also affects NW diameter. The average length of these NWs was ∼15 μm. A more detailed explanation of the role of temperature ramp-up rate on the NW diameter is explained in the following paragraph that details the NW growth mechanism.

**Figure 1:**
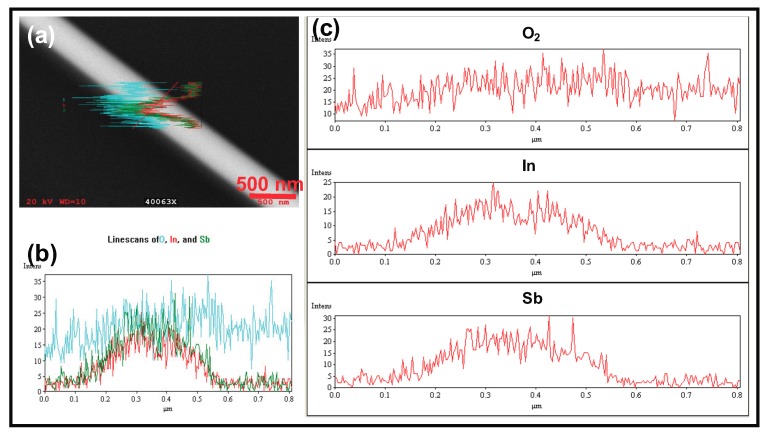
EDX spectrum showing composition mapping of a single InSb NW: (**a**) Intensity maps for In (red), Sb (green) and O${}_{2}$ (cyan) mapped across the width of the NW; (**b**) Intensity mapping of the three elements (O${}_{2}$, In and Sb) shown independently for comparison; (**c**) Superimposed elemental maps showing the NW is pure InSb. The relatively flat O${}_{2}$ EDX signal shows no characteristic feature corresponding to the NW and its origin is form the underlying SiO${}_{2}$ layer .

The role of sulfur in this work is that of a passivating agent [27]. Sulfur has a low solubility in the molten In seeds. It also has a high vapor pressure, which implies that at the growth temperature it is driven out of the growth region. This is why an EDX spectrum of the NWs did not show the presence of any sulfur. The addition of sulfur was critical to the growth process. An alternative would be to grow in 100% H${}_{2}$, but high concentration of H${}_{2}$ lowers the vapor pressure of Sb and so the resulting NWs are mostly just metallic In. Therefore, to maintain sufficient concentration of both In and Sb in the growing NW, it is important to have sulfur so that any trace of O${}_{2}$, can be removed from the growth region. To confirm that the NWs are purely InSb and there was no oxygen in the bulk of the NW, an EDX profile was obtained by performing a line scan in a direction perpendicular to the NW axis. As seen in Figure 1, the In and Sb signals (red and green curves) show maximum intensity at the center of the NW. The O${}_{2}$ signal (cyan) is relatively flat and has no characteristic feature across the width of the NW. Its presence is attributed to the fact that at 20 kV, the electron beam penetrates the InSb NW and the O${}_{2}$ signal arises from the 200 nm thick SiO${}_{2}$ layer that coats the Si substrate.

**Figure 2:**
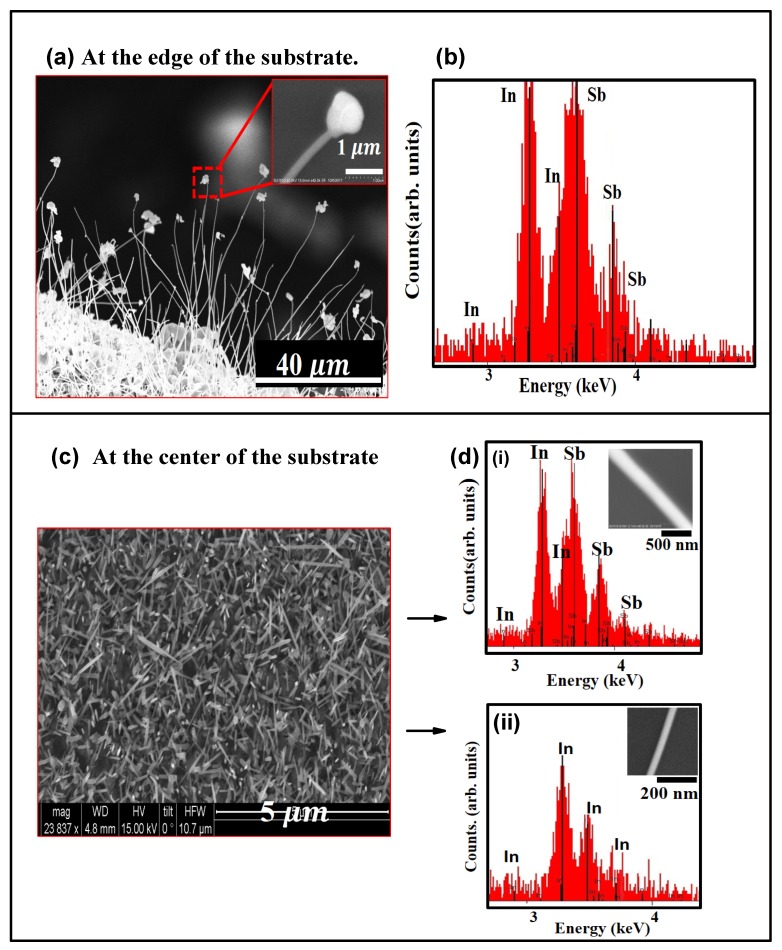
SEM image and EDX spectrum of the InSb NWs that are grown along the edge and at the center of the substrate: (**a**) SEM image showing a high density of NWs, growing from a bed of clusters or islands along the edge of the Si substrate. Inset shows a single NW with a highly deformed tip; (**b**) EDX spectrum of as grown NWs showing the NWs have a stoichiometric composition with In:Sb of 1:1; (**c**) SEM image showing a high density of NWs at the center of the Si substrate. There are no clusters or islands visible at the base of the NWs, as was evident in (**a**). The NWs grown in this region have different diameters and some were also found to have tapered ends; (**d**) NWs with diameter larger than 200 nm were InSb NWs, confirmed by the EDX spectrum shown in (**d-i**). NWs that have diameter less than 100 nm are In NWs, verified by the EDX spectrum shown in (**d-ii**).

**Figure 3:**
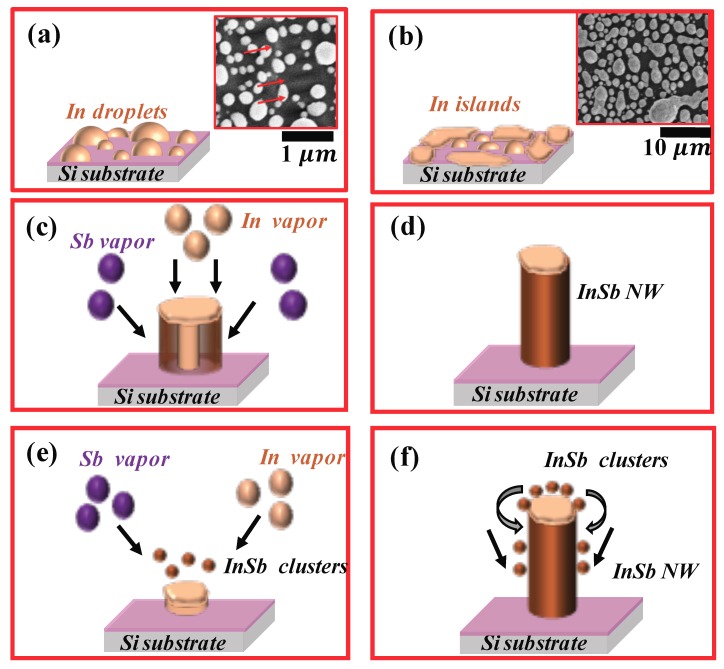
Schematic of the two possible growth mechanism of *a*-InSb NWs. (**a**) Initiation of NW growth as a result of In droplets forming on the Si substrate. These droplets have different sizes as shown in the inset; (**b**) As temperature rises at a very high rate, the droplets coalesce into islands and migrate to the edge of the Si substrate. Smaller In droplets are left behind at the center of the Si substrate; inset shows In islands coalescing and ripening; (**c**,**d**) Possible growth mechanism 1, in which an In core first precipitates from the molten seed (**c**), followed by complete antimonidization of the In core, resulting in an InSb NW (**d**); (**e**,**f**) Possible growth mechanism 2, in which In and Sb atoms combine to form InSb clusters over the molten In droplet (**e**). These clusters settle on the surface of the molten droplet and slips to the lower hemisphere and diffuses to the interface between the substrate and the molten In droplet (**f**). The mechanisms follow those reported for the growth of a-SiO${}_{x}$ NWs [28].

A notable feature of the experimental design is the high temperature ramp-up rate. The source temperature was increased at a rate of 75 ${}^{\circ }$C/min, until it reached the set point of 560 ${}^{\circ }$C. At such high temperature ramp up rates, the NW growth mechanism is most likely modified. Though growth most likely follows the VLS mechanism, it is possible that the high temperature ramp-rate affects the crystallinity of the growing NWs. SEM images depicting the morphology of the as-grown structures at the edge and at the center of the substrate is shown in Figure 2a,c respectively. During the initial stage of growth, as the temperature was raised up to the set point at the high rate of 75 ${}^{\circ }$C/min, the 200 nm thick In film breaks up into droplets. These droplets are not uniform in size and as seen in Figure 3a, the droplets range in diameter from about 100 nm to about 300 nm. As the temperature continues to increase, the In droplets increase in size by coalescing with other droplets nearby. These droplets have sufficient energy to migrate to the edge of the substrate where they are eventually immobilized as seen in Figure 3b. Therefore, the Si substrate has relatively large sized In-islands at the edges and smaller sized droplets at the center of the substrate. As seen in the SEM image shown in Figure 2a, the edges of the Si substrate has a high density of InSb NWs. The NWs grow off small nucleating sites on islands or clusters of InSb and most of them were found to have a deformed bulky tip, as seen in the inset of Figure 2a. As shown in the EDX spectrum of Figure 2b, the NWs are stoichiometric InSb with an In:Sb ratio of 1:1. Contrary to the deformed tips on NWs growing along the substrate edge, it was found that majority of the NWs on the body of the substrate did not have a tip. This is not in violation of the VLS NW growth mechanism, since NW growth was enabled by a self-seeded mechanism and it is possible that In was consumed during the growth process. An SEM image of the NWs grown at the center of the Si substrate (Figure 2c), shows that the NWs have different diameters, attributed to non-uniformity in the seed sizes (Figure 3a). It was also found that NWs with relatively large diameters (d > 200 nm) were mostly InSb (Figure 2d-i), while the thinner NWs at the center of the substrate (d < 100 nm) were mostly pure *In*, as seen in the EDX spectrum of Figure 2d-ii.

**Figure 4:**
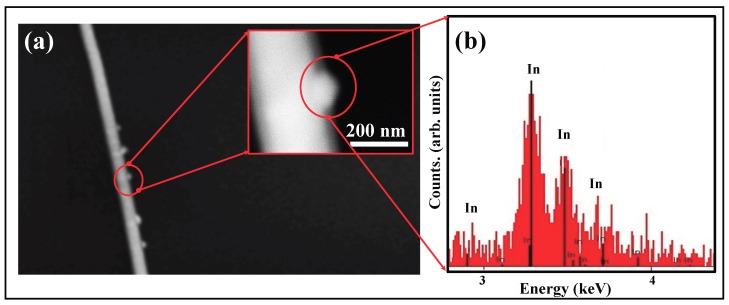
(**a**) SEM image of a single InSb NW grown at 560 ${}^{\circ }$C. Some defects are visible along the NW length and nodules are present along the NW edges. Inset shows a magnified view of the nodule grown along the NW edge; (**b**) EDX spectrum showing that the nodule is pure In.

Another significant feature of some of the NWs is shown in Figure 4a, where metallic In precipitates out of the growing NW and is present as `nodules’ on the edge of the NW. As seen in the EDX spectrum (Figure 4b), these nodules are pure In. Such precipitates of the metal catalyst along the NW edges have been reported by other groups. Their appearance is attributed to evaporation of the element In from the source and subsequent condensation along the NW edges, following the mechanism discussed in Refs. [29,30].

To explain the amorphous nature of the as-grown NWs, we consider the situation where as growth progresses, the temperature increases very rapidly to the growth temperature. The droplets receive incoming flux of In and Sb to reach the stage of supersaturation, from which the InSb solid phase precipitates out. The transformation from the liquid to solid phase requires some level of cooling of the molten droplet. As the solid NW phase begins to separate out from the highly disordered molten alloy, the droplet is cooled. Heat (q) is taken away from the droplet at the rate $\dot{q}$ [31], and the temperature (T) of the molten droplet decreases at a rate $\frac{dT}{dt}=\frac{\dot{q}}{mc_{p}}$ [31] where $c_{p}$ is heat capacity. The cooling rate of the molten alloy droplet determined by (dT/dt) is an indicator of the crystalline quality of the growing NW. The physical, chemical and thermodynamic condition of the molten droplet during this solidification phase will determine the arrangement of the atoms in the growing NW. The criteria for NW growth as expressed by the inequality equation between chemical potentials, $\mu_{v-l}^{l}>\mu_{l-s}^{l}$ [32] requires that the Gibbs free energy for a solid becomes less than that of the liquid ($G_{s}$<$G_{l}$). The difference in the Gibbs free energy provides the driving force for the liquid to solid phase transformation and is expressed as $\Delta G=G_{l}-G_{s}=\Delta H-T\Delta S$, where H is enthalpy of fusion and S is entropy in the system. At the temperature T, as the nucleating droplet begins to precipitate the solid phase as a NW, the arrangement of atoms in the nuclei is critical. The relatively high entropy of the nucleating site implies that there are movements of atoms in the tetrahedral arrangements, causing bonding and compositional changes in the growing NW. This rapid movement of In and Sb atoms in the solidifying (cooling) molten liquid is attributed to the high temperature ramp-rate and is the most likely cause for the amorphous nature of the InSb NW.

The exact mechanism wherein the *a*-InSb NWs solidifies from a molten catalyst is not very evident. A simple schematic of the InSb NW growth mechanism for the experimental conditions described above is shown in Figure 3. The process could follow either of the mechanisms discussed below and is based on the growth mechanism cited for synthesis of a-SiO${}_{x}$ NWs [28]. The first possible growth mechanism is based on the fact that due to the high volume of metallic In in the seed, an In NW initially precipitates out of the molten seed. As the temperature is rapidly raised to the growth temperature, Sb vapors impinge on the growing NW leading to antimonidization of the In NW. In this mechanism, the initially formed highly metallic In core is antimonidized; the antimonidization process continues as long as the In NW core exists. A simple schematic of the process is shown in Figure 3c,d There is a high possibility that antimonidization of an In NW core accounts for the synthesis of *a*-InSb NW, because when the Sb vapor pressure is reduced, the resulting NWs are pure metallic In (Figure 2d-ii). A second possible mechanism that results in the formation of *a*-InSb NWs is the case where the Sb does not penetrate the In droplet. Instead, InSb clusters form over the molten In droplet. Due to the low solubility of InSb in In , the InSb clusters slip to the lower hemisphere of the molten catalyst and begins to grow as a 1-D NW. Continuous supply of InSb clusters then support the growth of an *a*-InSb NW as shown in Figure 3e,f.

**Figure 5:**
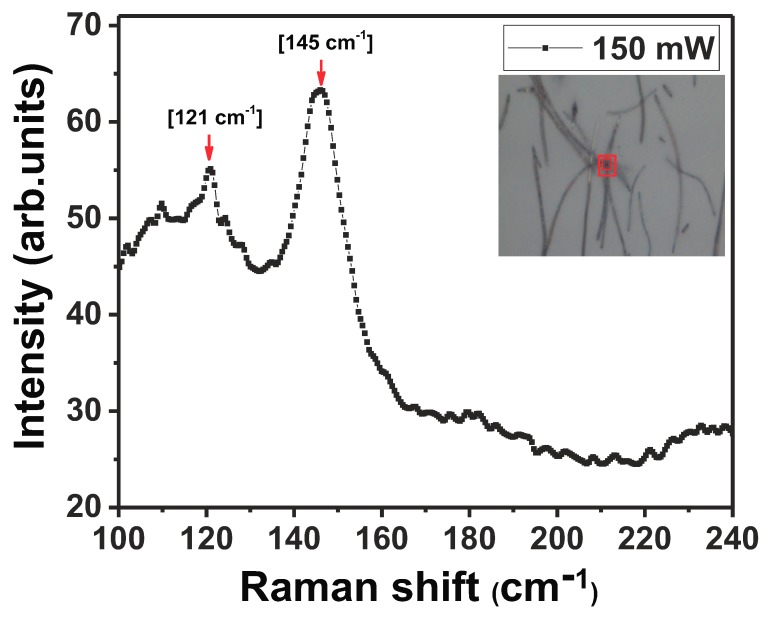
Raman spectrum obtained from a single *a*-InSb NWs at room temperature. Inset shows an optical image of the region (crossed NWs) from where the Raman spectrum was collected. The two peaks at ∼145 cm${}^{-1}$ and 121 cm${}^{-1}$ are attributed to *a*-InSb. The spectrum was obtained with 150 mW of laser power.

**Figure 6:**
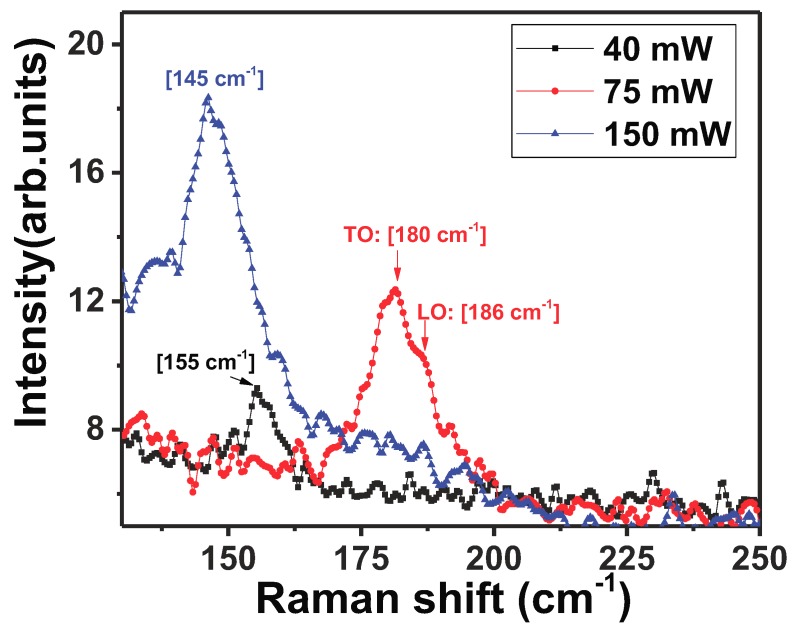
Comparison of Raman spectrum of a single *a*-InSb NWs, measured with increasing laser power on the sample. The laser power was varied from 40 mW to 150 mW. At 40 mW, the broad peak around ∼155 cm${}^{-1}$ is attributed to homopolar Sb-Sb bonds in *a*-InSb. Increasing laser power (75 mW), causes localized crystallization of the NW, resulting in characteristic crystalline peaks measured at 181 cm${}^{-1}$ and at 186 cm${}^{-1}$. Further increase in the laser power results in reverting the material back to its amorphous sate. This is evident in the disappearance of the crystalline peaks and the emergence of the broad peak around ∼145 cm${}^{-1}$.

Raman spectrum of *c*-InSb is well documented and is known to be characterized by TO phonon 179.8 cm${}^{-1}$ and LO phonon modes 190.8 cm${}^{-1}$ [6,10,33,34,35,36]. The amorphous nature of a single as-grown InSb NW was first verified by Raman spectroscopy studies at room temperature (Figure 5). The stoichiometric NW (In:46.9 wt%, Sb: 53.1 wt%) exhibited a Raman spectrum with a relatively wide peak at low frequency, centered around ∼145 cm${}^{-1}$. This peak has been attributed to a high density of homopolar Sb-Sb bonds that are found in *a*-InSb [37,38,39]. The aggregation of Sb-Sb defects is attributed to growth conditions which include NW growth in an Sb-rich environment and a high temperature ramp-up rate. Additionally, a shoulder peak was detected around 120 cm${}^{-1}$. This peak was observed in several amorphous semiconductors like *a*-Si, *a*-Ge, *a*-GaAs and *a*-InSb [39,40], and is reported to occur near the LA branch of a crystal. To study the effect of laser light on the amorphous nature of the as-grown InSb NWs, the evolution of the Raman spectrum as a function of laser power was studied at room temperature, by measuring the Raman spectrum at the same spot on the NW for three different laser intensities. The results are shown in Figure 6. The broad peak around ∼155 cm${}^{-1}$, attributed to the presence of Sb-Sb bonds is evident at the low laser power of 40 mW. The laser beam was focused at a spot on the NW for 60 s. As the laser power was increased to 75 mW, the peak attributed to *c*-InSb at 181 cm${}^{-1}$ (TO phonon) and at 186 cm${}^{-1}$ (LO phonon) appear as shown in Figure 6. The observation of these peaks indicates that the laser light induced localized crystallization of the area hit by the laser beam. Further increase in the laser intensity causes the crystalline peaks at 181 cm${}^{-1}$ and 186 cm${}^{-1}$ to disappear and the peak attributed to *a*-InSb re-appears around 145 cm${}^{-1}$. It is thus evident that with increasing laser power, structural defects of the Sb-Sb type form, returning the crystal to its amorphous state. The slight shift (about 10 cm${}^{-1}$) in the position of the peaks in *a*-InSb is most likely induced by stress in the highly defective material. Laser light intensity is thus shown to cause radiation-stimulated diffusion of defects in *a*-InSb.

**Figure 7:**
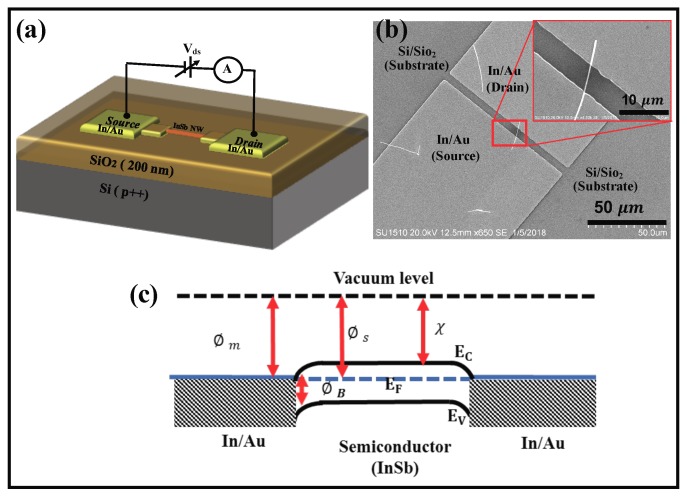
(**a**) A schematic of the two-terminal device showing the single NW with the In/Au source and drain contacts. (**b**) An SEM image of a single InSb NW contacted by In/Au contacts; inset shows an enlarged view of the channel that contained a single InSb NW. (**c**) A schematic of simplified energy band diagram of the metal-nanowire-metal contact in the absence of any applied bias.

**Figure 8:**
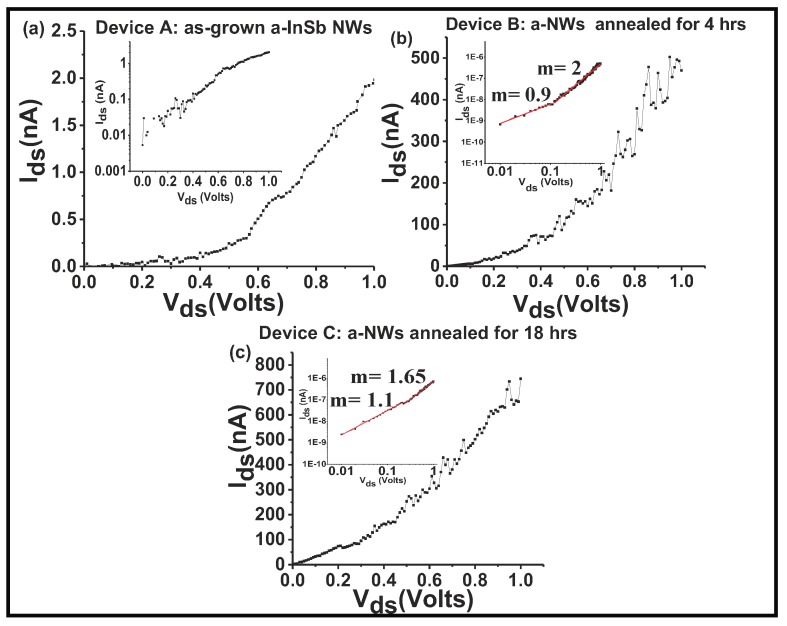
Current-Voltage curves of three devices comprising of single *a*-InSb NW, contacted by In-Au electrodes. (**a**) I-V curve of Device A showing non-linear behavior; the semi-log plot in the inset also shows a non-linear trend indicating that the contact is not a Schottky contact; (**b**) non-linear I-V curve of Device B; inset shows a linear log-log plot with two different slopes in the low bias (*m* = 0.9) and high bias regime (*m*= 2); (**c**) I-V plot of Device C showing the curve is becoming more linear as the material becomes more crystalline with long-duration anneal. At low bias, all three plots obey Ohm’s law. At high bias voltage, the plots follow Iα$V^{m}$ where the values of m varying from 3.1 to 1.6. The decreasing values of m as the InSb NWs are annealed for longer time periods verifies the efficacy of heat treatment for transforming *a*-InSb to *c*-InSb.

To ascertain the possibility that thermally activated defect diffusion is also a possibility, *a*-InSb NWs were subjected to post-growth heat treatments and their electronic quality was assessed by analyzing their current-voltage behavior. In the first set of experiments, as-grown *a*-InSb NWs were annealed in a tube furnace for 4 hrs under flow of Ar gas at 150 ${}^{\circ }$C. Raman spectrum showed that the NWs retained their amorphous nature. In the second set of experiments, the as-grown *a*-InSb NWs were annealed in an inert environment at 150 ${}^{\circ }$C for 18 hrs. Current-voltage response of the three devices [Device A (as-grown *a*-InSb NWs); Device B (*a*-InSb NWs after 4 hrs of thermal anneal at 150 ${}^{\circ }$C); and Device C (*a*-InSb NWs after 18 hrs of thermal anneal at 150 ${}^{\circ }$C)] was analyzed. The device schematic is shown in Figure 7a. Figure 7b is an SEM image of the contacted NW; inset shows an enlarged view of the channel that contained a single InSb NW. The channel length was determined to be 5 μm. The magnitude of the current through the device depends on the characteristic of the metal-semiconductor contact and the intrinsic properties of the *a*-InSb. Due to the device geometry, the effective area of the metal contact with the NW is ≈10${}^{-12}$ m${}^{2}$, corresponding to the total metal-semiconductor contact area. Since the two contacts to the NW are the same (In/Au contacts), a simplified energy band diagram of the metal-nanowire-metal contact in the absence of any applied bias is as shown in Figure 7c, where the *a*-InSb is considered to be n-type. Considering that the metal contact is In/Au (defined by a work function of 5.1 <$\Phi_{m}$< 4.09), the metal-semiconductor contact is characterized by the case where the metal work function is less than that of the semiconductor. Though the work function of *a*-InSb is not documented, assuming a work function close to *c*-InSb (4.57 eV), it is possible that the barrier height at both the interfaces are negative and the contacts are ohmic. The magnitude of the current through the device would therefore be determined by the resistance of the nanowire. To validate the hypothesis that the contacts are ohmic, the non-linear I–V plots (Figure 8a–c) recorded at room temperature were analyzed. The I–V data for device A (as-grown NWs) was first studied to assess a possible Schottky diode behavior by creating a semi-log plot of the I–V curve. For a Schottky diode behavior, the semi-log plot of I–V should exhibit linear characteristics. The non-linearity of the I–V curve (shown in the inset of Figure 8a) confirms that the metal-semiconductor contact is not a Schottky contact. Moreover, the ideality factor (*n*), estimated from the fitting parameter q/nkT gives n much grater than 100, whereas n=1 for an ideal Schottky junction. It was hence concluded that the non-linearity in the I–V curves is not due to current through a Schottky junction.

There are three fundamental carrier injection processes: (i) conduction by thermionic emission, (ii) conduction by space-charge-limited-current (SCLC), and (iii) Fowler-Nordheim (F-N) tunneling induced conduction. To ascertain a power dependence, a log-log plot of the I-V curves was analyzed (Figure 8b,c). For all three devices, in the low-bias regime the I-V plot is linear (m ≈ 1), which implies that in the voltage ranges below ≈ 0.4 V, the conduction mechanism is related to thermionic emission type of conduction, and for all three devices, in the low-bias regime, I ∝ V and the device obeys Ohm’s law. The linear fit to the log-log plots for all three devices for bias above 0.4 V shows values of slope varying from 3.1 to 1.6, indicating that in this relatively high-bias regime, I ∝$V^{m}$, with m decreasing as the NW device is annealed for longer durations. For the as-grown NW device, *m* = 3.2, while for NW that was annealed for 4 hrs the value of m decreases to 2 and for the NW that was annealed for 18 hours, m decreases further to 1.6. The high values of m are consistent with the space-charge-limited current (SCLC) conduction model [20,41]. A possible explanation for different transport mechanisms in the low and high bias regimes and for the changing m is the existence of a high density of defects at the metal-semiconductor interface. These defects can trap injected electrons and increase the electric field at the interface. After 18 hrs of annealing, the 50% reduction in the value of m indicates that the NW is becoming more crystalline and the conduction becoming more ohmic. A comparison of the NW resistivity shows that the as-grown sample has a high resistivity of ∼19 × 10${}^{2}$± 4 ×$10^{2}$Ω cm. For the InSb NWs that were annealed at 150 ${}^{\circ }$C for 4 hrs, the resistivity drops to about 7 ± 2 Ω cm and the NW that was annealed for 18 hrs has a resistivity of 2 ± 0.2 Ω cm. These values are about 10 times higher than that reported for *c*-InSb NWs that have a resistivity of about 0.3 Ω cm [42]. These results indicate that the electrical properties of the *a*-InSb NWs are related to the quality of the NWs and also to the presence of defect states. At low bias fields, the mechanism of carrier transport is found to be via thermionic field emission. However, at high bias, the interface defect states modify the charge transport mechanism and conduction occurs via the SCLC.

## 4. Conclusions

A new and relatively simple technique to synthesize *a*-InSb NWs and to transform them post-growth into crystalline NWs is presented in this paper. The relatively high temperature ramp-up rate during NW growth is believed to be the main factor affecting the crystalline quality of the NW. Laser light-induced radiation effects and post-growth heating effects were both found to affect the crystalline quality of the as-grown NWs. The non-linearity of the current-voltage measurements on individual NWs is attributed to different conduction mechanisms in the low and high-bias regions. Post-growth annealing was found to be effective in transforming the amorphous NW into a crystalline one with improved electrical conductivity. Further studies are required to ascertain the role of traps and defects in the power-law dependence observed in the current-voltage measurements.
